# Data on changes of NF-κB gene expression in liver and lungs as a biomarker and hepatic injury in CLP-induced septic rats

**DOI:** 10.1016/j.dib.2019.104117

**Published:** 2019-06-18

**Authors:** Saba Miri, Azadeh Rasooli, Satinder Kaur Brar

**Affiliations:** a INRS-ETE, Université du Québec, 490, Rue de la Couronne, Québec, G1K 9A9, Canada; b Department of Biochemistry, Faculty of Sciences, Payame-e-Noor University, Tehran, Iran; c Department of Civil Engineering, Lassonde School of Engineering, York University, North York, Toronto, Ontario M3J1P3, Canada

**Keywords:** Sepsis, Inflammation, NF-κB, Gene expression, Hepatic cell evaluation

## Abstract

Nuclear factor kappa-light-chain-enhancer of activated B cells (NF-κB) is a ubiquitous transcription factor, which plays a key role in regulating immune response against infection. Increased and/or prolonged activation of NF-κB has been linked to cancer, inflammatory, autoimmune diseases and viral infection. The purpose of this set of data was to evaluate NF-κB gene expression in cecal ligation and puncture (CLP)- induced septic rats and associated hepatic cell changes. Here, we provide the information about the evaluation of NF-κB gene expression using Real-time PCR and histopathological data of liver-related to our study published in the Turkish Journal of Medical Sciences [1]. Also, the information of the primers and more details about gene expression evaluation by real-time PCR of the targeted gene is provided. The data present here introduced another biomarker in liver and lung of CLP- induced septic rats and also confirmed hepatic dysfunction based on the pathological data. The histopathologic assessment showed normal condition in control group, mild infiltration of neutrophils in the liver parenchyma and portal tracts in LAP group (laparotomy) but severe congestion, severe neutrophil infiltration in the liver parenchyma and portal tracts in the CLP group. The data from real-time PCR showed that NF-κB expression was significantly increased in the CLP group compared with the control and LAP group, so it can be a biomarker for (CLP)- induced sepsis. This set of data and our previous study underscored the powerful potential of using the real-time PCR method to determine the involvement of genes such as MPO, CD177, and NF-κB in infectious diseases like sepsis.

**Table:** Specifications table

Subject area	Medical Sciences/biology
More specific subject area	Inflammatory disease, molecular biology
Type of data	Table (Temperature condition and primers), image (pathological evaluation), figures (real-time PCR)
How data was acquired	Primers for real-time PCR were designed using Primer3, a PCR primer design program, and BLASTN searches were used to check primer specificity. Real-time PCR: Rotor-Gene Q system (QIAGEN, Germany) Pathological data: light microscopy (Olympus CX31RBSF)
Data format	Raw and analyzed
Experimental factors	Male Wistar rats were divided into three groups of control (without any surgery), laparotomy (LAP), and cecal ligation and puncture (CLP). The data here is given for gene expression levels of liver and lung tissue from these groups. Data for histopathology evaluation were investigated for liver tissue.
Experimental features	The effect of sepsis on liver and lung tissue were investigated. NF-κB expression was investigated as an inflammatory gene in liver and lung tissue of control, LAP and CLP group. The liver specimens of all rats were examined under light microscopy to assess the hepatic changes.
Data source location	Department of Biochemistry, Faculty of Sciences, Payame-e-Noor University, Tehran, Iran
Data accessibility	Data presented in this article.
Related research article	A. Rasooli, E. Ghafari, H. SAEIDI, S. Miri, Expression changes of CD177 and MPO as novel biomarkers in lung tissue of CLP model rats, Turkish journal of medical sciences, 48 (6), 2018, 1321–1327 [1].

**Table:** 

**Valdue of the data** ∙ The data set can be referenced by other researchers investigating the effect of sepsis on liver and lung tissue. ∙ The data from the NF-κB expression level as an inflammatory gene can be a potential biomarker for sepsis. ∙ The data from histopathological evaluation provides a clearer understanding of the hepatic cell changes in sepsis.

## 1 Data

The detailed information of real-time PCR condition used in this data set and our previous is presented in Table 1.

**Table 1:** Temperature condition and primers used in the PCR and Real-time PCR.

Step		Experimental temperature and primer sequence		Time for each step	Cycle
Initial Denaturation		95 °C		15min	1
PCR	Denaturation	95 °C		15sec	40
	Annealing	59 °C for NF-κB primers	Forward NF-κB: 5′-CGCAAAAGGACCTACGAGAC-3′ Reverse NF-κB: 5′-TGGGGGAAAACTCATCAAAG-3′	20sec	
		60 °C for GAPDH primers	Forward GAPDH: 5′-TGCCAGCCTCGTCTCATAG-3′ Reverse GAPDH: 5′-ACTGTGCCGTTGAACTTGC-3′		
	extension	72 °C		20sec	
Melt Curve		57–95 °C (raising by 0.3 °C)		15sec for each step	1

**Fig. 1:**
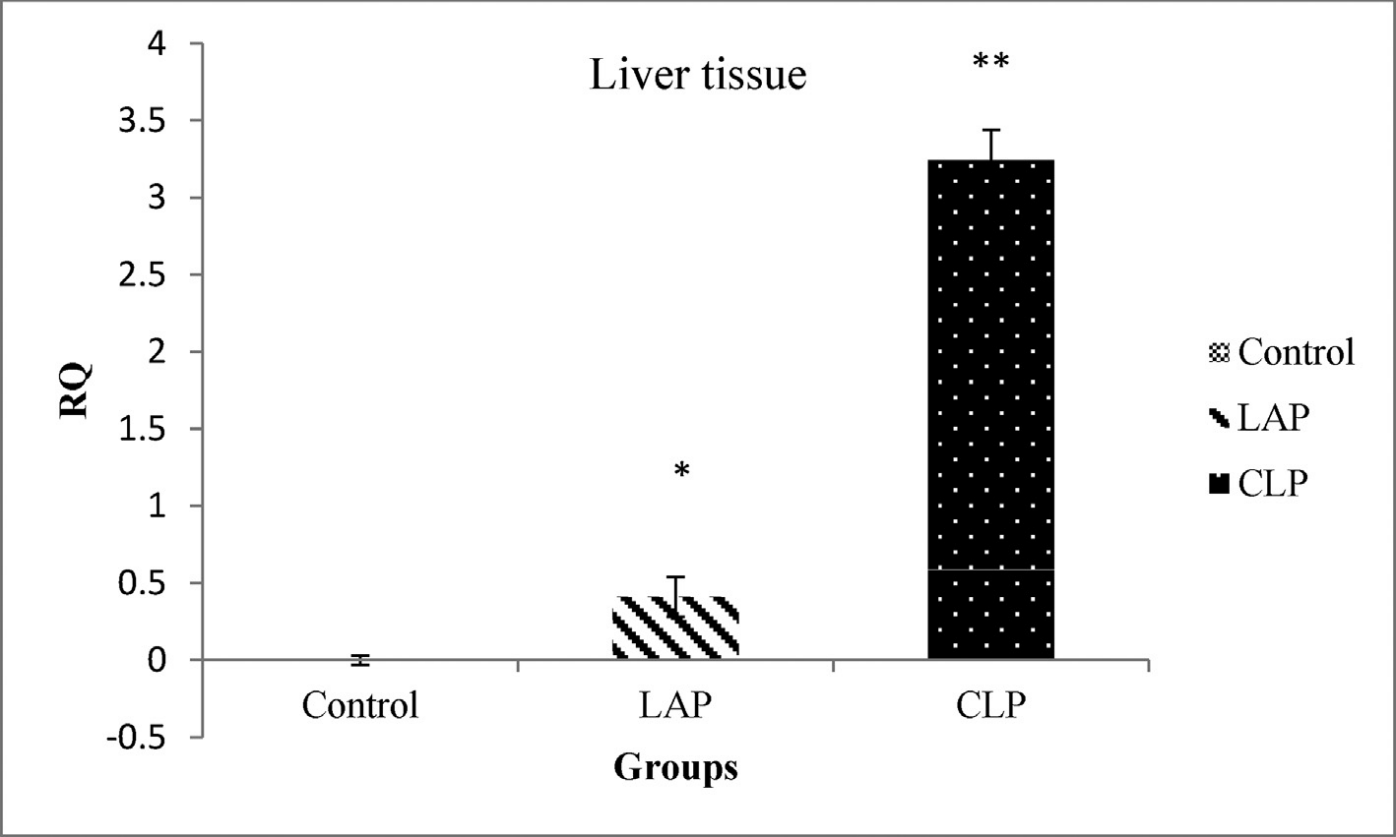
The gene expression of NF-κB in liver tissues. *P < 0.05 is considered significantly between the control group and LAP group. **P < 0.05 is considered significantly between LAP group and the CLP group. Data are presented as mean ± SD.

**Fig. 2:**
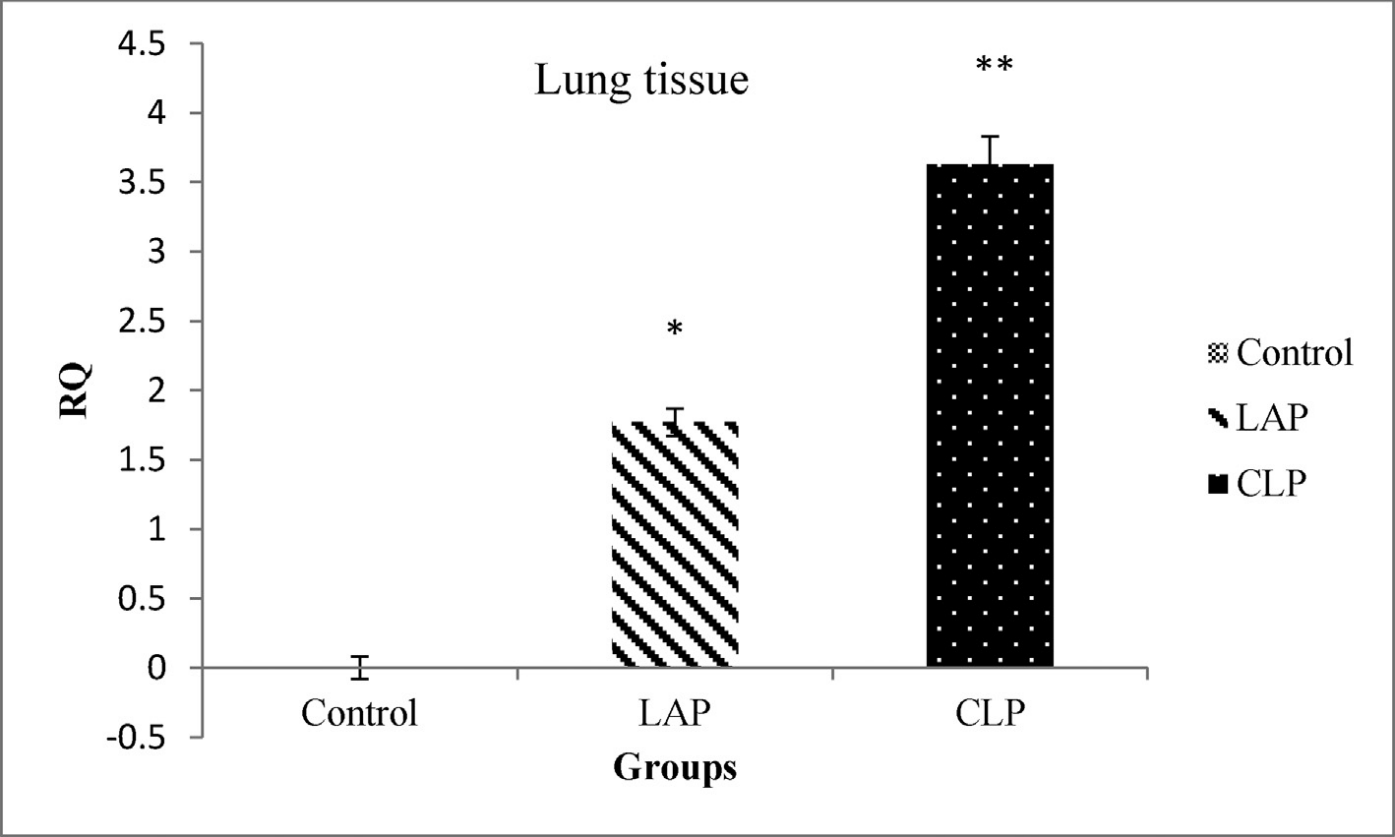
The gene expression of NF-κB in lung tissues. *P < 0.05 is considered significantly between the control group and the LAP group. **P < 0.05 is considered significantly between LAP group and the CLP group. Data are presented as mean ± SD.

Fig. 1, Fig. 2 shows the normalized data of NF-κB expression from real-time PCR in three groups of control, LAP, and CLP in liver and lung respectively. As shown in Fig. 1, Fig. 2, levels of gene expression of NF-κB were markedly increased in the CLP group compared with LAP and control group (P < 0.05).

Also, it was observed that laparotomy as shock surgery could elevate the level of NF-κB gene expression when compared with the control group (P < 0.05).

**Fig. 3:**
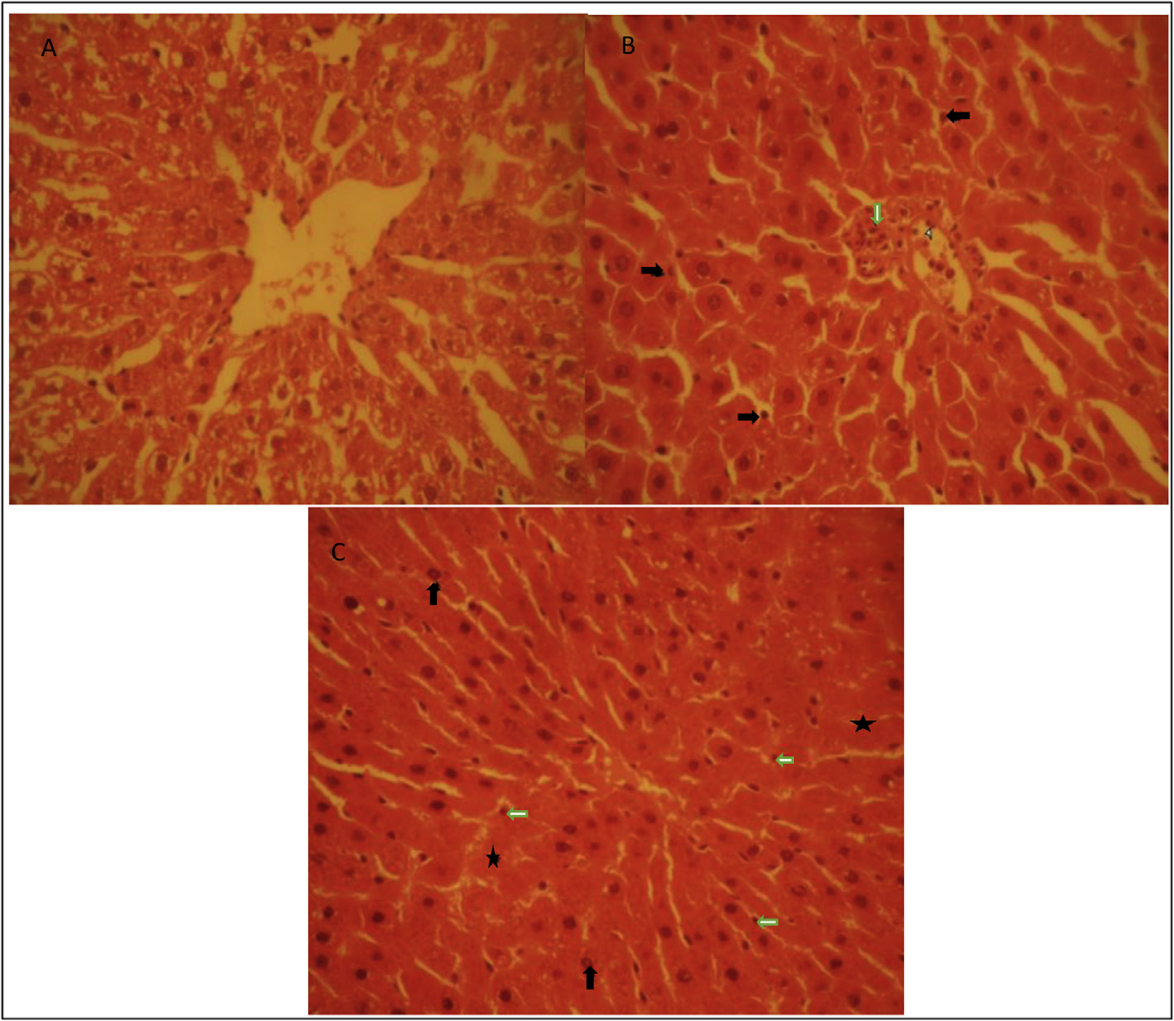
Histopathological studies. A) Control group B) LAP group C) CLP group. Infiltration of neutrophils in the liver parenchyma and portal tract showed with black arrows and white arrow respectively. Also, necrotic hepatocytes with pyknotic or karyolytic nuclei sowed with stars.

The histopathologic assessment showed that the liver in the control group was in normal condition (Fig. 3A). In the LAP group, there was mild infiltration of neutrophils in the liver parenchyma (black arrows) and portal tracts (white arrow). Also, neutrophil margination could be seen in the vein (arrowhead) (Fig. 3B). Whereas, severe congestion, severe neutrophil infiltration (white arrows) in the liver parenchyma and portal tracts were observed in the CLP group (Fig. 3C). Hepatic cells show degenerations including chromatin clumping and nuclear vacuolation (black arrows). Necrotic hepatocytes with pyknotic or karyolytic nuclei (stars) are obvious in the picture as well (Fig. 3C).

## 2 Experimental design, materials and methods

### 2.1 Experimental animals

Female Wistar rats (120–150 g) were used in the present data set. Rats were procured from Pasteur Institute. These animals were kept in room temperature 22–23 °C; relative humidity 50%; 12-h light-dark cycle and fresh drinking water was offered to animals ‘daily *ad-libitum.* The experiments were carried out in accordance with the guidelines of the Animal Ethics Committee, the World Medical Association Declaration of Helsinki (Adopted by the 18th World Medical Assembly, Helsinki, Finland, June 1964). Animals were divided into three groups containing 10 in each group as a control, laparotomy (LAP) and cecal ligation and puncture (CLP) groups. After 48 h of CLP surgery, animals were anesthetized with chloroform, liver and lung tissues were removed for further experiments.

### 2.2 Sepsis induction by CLP

Sepsis was induced using CLP according to the previously described method by Rasooli et al. (2018).

Animals were anesthetized with an intraperitoneal injection of a mixture of 10 mg/kg xylazine and 90 mg/kg ketamine hydrochloride. 2-3-cm midline laparotomy was made on the anterior abdomen and the caecum was exposed and ligated by silk 4-0 at distal of the ileocecal valve, without causing intestinal obstruction. The cecum was punctured twice with a 20G needle. The cecum was repositioned, and the incision was closed with 3-0 suture material. All rats were allowed free access to food and water after CLP. For LAP groups, all the CLP procedures were performed except the ligation and perforation of cecum [2].

### 2.3 RNA isolation and cDNA synthesis

Total RNA was isolated from the liver and lung tissues using the RNA total kit (BioBasic BS584, Canada) as described by the manufacturer's instruction. The isolated RNA was treated by DNase I (Sinaloa Co., Tehran, Iran), and the concentration of RNA was determined by absorbance at 260 nm using Nanodrop 2000 spectrophotometer (Thermo Scientific). The same amount of RNA (1μL) was used for each cDNA reaction. Reverse transcription reaction was carried out with cDNA Synthesis Kit (Takara Shuzo). Reverse transcription was performed at 37 °C for 15 min and 85 °C for 5 min. cDNA was stored at - 20 °C until use.

### 2.4 Real-time PCR

Quantitative real-time PCR was performed on the cDNA samples using SYBRR Green Real-time PCR Master Mix (Rotor-Gene Q- QIAGEN) in Rotor-Gene system (Rotor-Gene Q- QIAGEN). Primers were designed for real-time PCR with Primer3 [3], then primer specificity was further tested by computer-based analysis against databases with BLASTN (Table 1). The amount of the target gene was normalized first to the endogenous reference (GAPDH) and then relative to a calibrator (sample with control animal); the fold changes were analyzed by the comparative C(T) method by using the formula 2^−ΔΔCt^ as following Equations (1), (2), (3)):

(1) ΔCt _(surgery group)_ = Ct _(NF-κB)_ – Ct _(GAPDH)_

(2) ΔΔCt = ΔCt _(surgery group)_ – ΔCt _(control group)_

(3) $$\text{Fold}\;\text{change}=2^{-\text{ΔΔCt}}=\frac{1}{2^{\text{ΔΔCt}}}$$

### 2.5 Histopathology

The liver specimens of all rats were fixed in 10% buffered neutral formalin solution and washed with tap water for at least 3 h, then by increasing concentrations of alcohol, the samples were gradually dehydrated. The tissue submerged in paraffin and embedded in paraffin blocks. Tissue samples were sectioned 5-6 μm-thick and placed on slides. To remove the paraffin, the sections were kept in toluene for 2 h. After staining with hematoxylin and eosin (H&E), the sections were washed twice with toluene and covered with Entellan [4] then examined under light microscopy (Olympus CX31RBSF) to assess the hepatic changes.

## Conflict of Interest

The authors declare that they have no known competing financial interests or personal relationships that could have appeared to influence the work reported in this paper.
